# Experimental data regarding the characterization of the flame behavior near lean blowout in a non-premixed liquid fuel burner

**DOI:** 10.1016/j.dib.2015.11.051

**Published:** 2015-12-08

**Authors:** Maria Grazia De Giorgi, Aldebara Sciolti, Stefano Campilongo, Antonio Ficarella

**Affiliations:** University of Salento, Italy

**Keywords:** Combustion, Flame, VIS-CCD, NIR-CCD, PMT

## Abstract

The article presents the data related to the flame acquisitions in a liquid-fuel gas turbine derived burner operating in non-premixed mode under three different equivalence fuel/air ratio, which corresponds to a richer, an intermediate, and an ultra-lean condition, near lean blowout (LBO).

The data were collected with two high speed visualization systems which acquired in the visible (VIS) and in the infrared (NIR) spectral region. Furthermore chemiluminescence measurements, which have been performed with a photomultiplier (PMT), equipped with an OH* filter, and gas exhaust measurements were also given. For each acquisition the data were related to operating parameters as pressure, temperature and equivalent fuel/air ratio.

The data are related to the research article “Image processing for the characterization of flame stability in a non-premixed liquid fuel burner near lean blowout” in Aerospace Science and Technology [1].

**Specifications Table**

**Table:** 

Subject area	Engineering
More specific subject area	Combustion, gas turbine
Type of data	Table, image, figure
How data was acquired	VIS High rate CCD: MEMRECAM GX-3® of NAC Image Technology
NIR High rate CCD: FLIR A2600sc NIR camera
Photomultiplier with OH* filter and data acquisition system: PMTSS of Thorlabs®, filter at (306.4±5) nm, DAQ device of National Instruments® NI-USB 2008
Pollutant emissions analyzer: PG-350E Horiba
Fuel mass flow meter: VSE 0.02 flow meter
Air mass flow calculated by the Labview® control platform using the data measured by the pressure sensor Nuova Fima ST18 and by a t T-type thermocouple.
Data format	Raw data of the pixel intensity matrix of the flame images, of photomultiplier acquisition, of pollutant emissions and control platform
Experimental factors	Controlled temperature, pressure and fuel/air ratio
Experimental features	Experiments were realized at atmospheric pressure and preheating the air up to 500 K. Fuel/air ratio was fixed at different values and stabilized for 10 min before acquiring all the signals simultaneously.
Data source location	Green Engine Laboratory, Department of Engineering for Innovation, University of Salento-Lecce (Italy).
Data accessibility	The data are provided in supplementary files directly with this article.

**Value of the data**

• In the literature few experimental data are available for liquid-fuel gas turbine derived burner.
• Investigation of flame behavior near lean blowout is essential for the development of novel environmental friendly combustors. The identification of blowout precursor to be used for the development of a real time control system is essential to avoid the damage due to LBO.
• The provided data are suitable for comparing the burner with others characterized by different geometry and/or operative conditions.
• These experimental data permit to calibrate/validate numerical CFD modeling approaches for combustion and instabilities fields.

## 1 Data

Data in this paper are presented for three values of fuel/air ratios: a richer (*Φ*=0.333), an intermediate (*Φ*=0.212), and an ultra-lean condition (*Φ*=0.144), in Supplementary files in the following series:

• Photomultiplier signals, which were acquired at 5 kHz for an acquisition time of 1 s. The table of 5000 values for three *Φ* are reported in the file “CONTROL_PMT_EMISSIONS”, sheet “PMT” in the Supplementary material.
• Pollutant emissions data: NO_*x*_, CO_2_, CO, O_2_, SO_2_. They are recorded with a frequency of 1 Hz for 1 s of acquisition time. The single value of each species for the three values of fuel/air ratio are reported in the file “CONTROL_PMT_EMISSIONS”, sheet “POLLUTANTS” in the Supplementary material.
• Control platform data: air mass flow, fuel mass flow, air inlet temperature, fuel inlet temperature, 3 values of temperature in the combustion chamber, combustion chamber pressure. The platform acquires at 4 Hz and the data acquired in 1 s are reported. They are reported in the file “CONTROL_PMT_EMISSIONS”, sheet “CONTROL PLATFORM” in the Supplementary material.
• Luminosity matrix of the VIS images. The images were taken at 10 kHz for 1 s of acquisition time. The resolution is 288×384 (0.31 mm/pixel). Here only 1000 images are reported. Two 3D matrix of 288×384 elements and 1000 sheets are reported in the following Matlab workspaces of the supplementary material: “VIS_0_333.mat”, “VIS_0_212.mat”, “VIS_0_144.mat”. The number in the file name represents the equivalent fuel/air.
• Luminosity matrix of the NIR images. The images were taken at 1024 Hz for 1 s of acquisition time. The resolution is 64×64 (0.175 mm/pixel). The 3D matrix of 64×64 elements and 1024 sheets is reported in the following Matlab workspaces of the supplementary material: “NIR_0_144”, “NIR_0_212”, “NIR_0_333” of the Matlab workspace Supplementary material. The number in the file name represents the equivalent fuel/air.

**Fig. 1:**
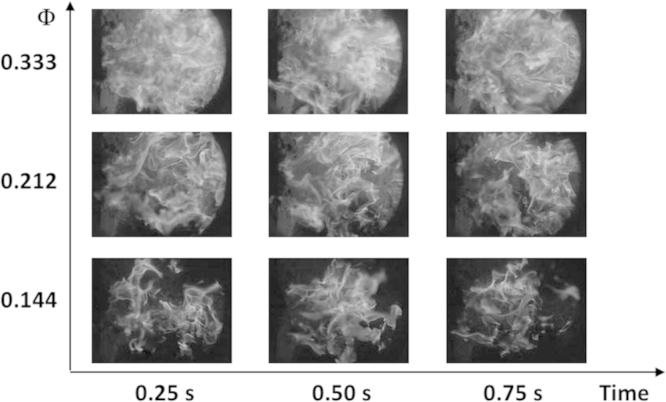
Example of VIS raw images for different values of *Φ* and in different time steps.

An example of the VIS raw images are also directly provided with in the text of this article (see Fig. 1).

## 2 Experimental design, materials and methods

**Fig. 2:**
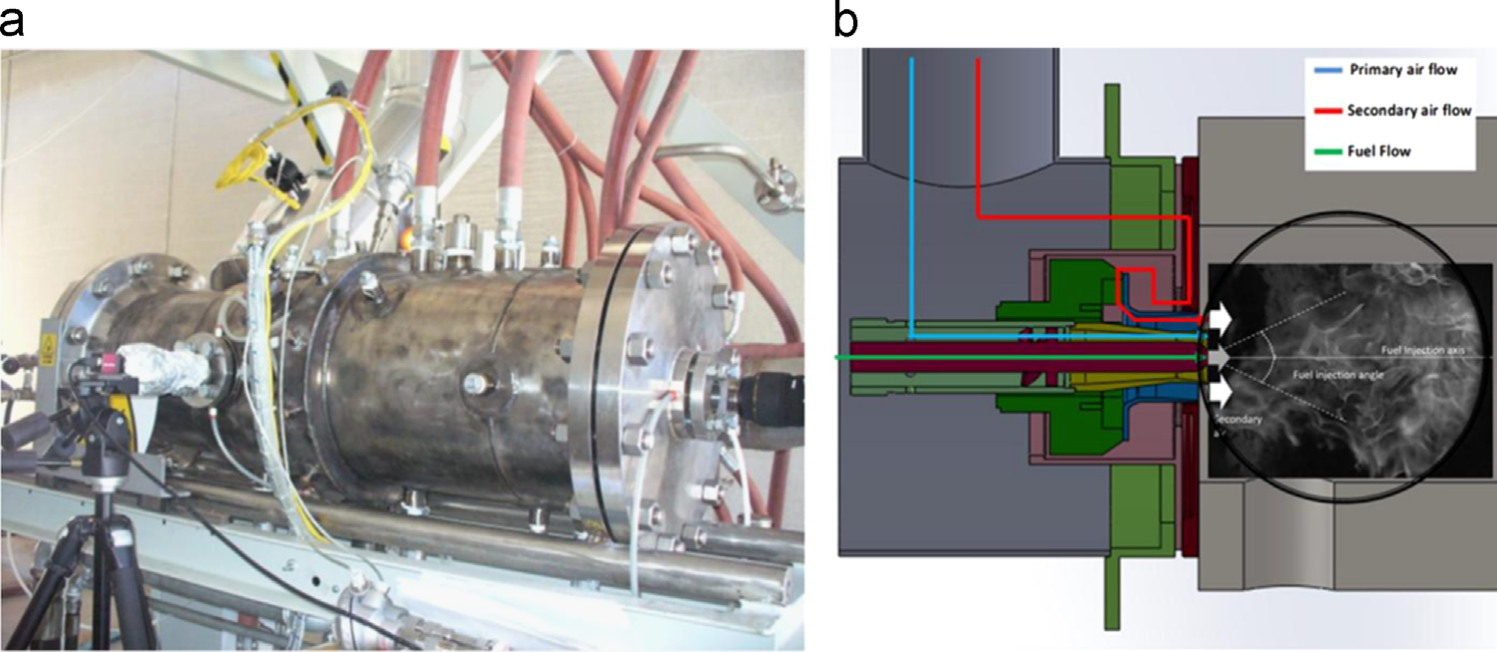
(a) The Green Engine burner; (b) sketch of the burner chamber and injection line with the indication of the air and fuel paths in non-premixed combustion mode.

The experiments were carried out using the combustion test rig at the Green Engine laboratory of the University of Salento in Lecce – Italy, where a 300 kW liquid-fueled swirling combustor was used (Fig. 2a). The burner is a gas-turbine derived combustor, modified for research׳s investigations [[2], [3], [4], [5]. Fig. 2b shows the geometry of the burner chamber and fueling system. The inner diameter of the burner is 14 cm and its length is 29 cm. The air passage consists of two concentric annular air tubes. The inner one is equipped with eight-septa, 45° swirler. At the exit, the combustion chamber contracts to a cylindrical exhaust gas nozzle.

The burner can be operated by non-premixed or partially premixed mode fuel injection, and it permits adjustable settings, allowing the study of a wide range of flame types. In the present study the non-premixed combustion mode has been investigated and in Fig. 2b the air flow path is shown. Liquid fuel is supplied by a gear pump with a rotation speed of 900 rpm and it can be pressurized to an absolute pressure up to 10×10^5^ Pa. Liquid fuel enters the injector which is equipped with a single-hole nozzle by Monarch, BPS 3GPH@100 psi line injection (3.69×10^−6^ m^3^/s @ 7 bar line injection) which is characterized by an injection angle of 45°.

For the control of the combustor the National Instruments LabVIEW® integrate platform was used. The mass flow rate of fuel is measured using the VSE 0.02 flow meter that specifies measurement accuracy of up to 0.3% of the measurement value and a repetition accuracy of 0.05%. The air mass flow is calculated using the pressure measurement obtained using the pressure sensor Nuova Fima ST18 (uncertainty ±0.5% of reading) and the temperature measurements at the air inlet (uncertainty ±1% of reading): hence the max combined uncertainty on the air mass flow is 1.7%.

In each operative condition the 8.5% of the air flow follows the blue way (primary air) while the major quantity of the air enters in the swirler following the red path (secondary air).

The combustor is equipped with optical accesses that permits to capture the flame emissions in the visible spectral range emission by using the high speed CCD camera MEMRECAM GX-3® of NAC Image Technology [6], in the near infrared spectral range through the FLIR A2600sc NIR camera [7] and also the chemiluminescence emissions of OH* captured by using the photomultiplier PMTSS of Thorlabs® [8] (with a (306.4±5) nm interference filter) using the data acquisition (DAQ) device of National Instruments® NI-USB 2008 [9] (5 kHz, 1 s of acquisition time for each test). The CCD cameras were located perpendicularly to the flame axis.

**Fig. 3:**
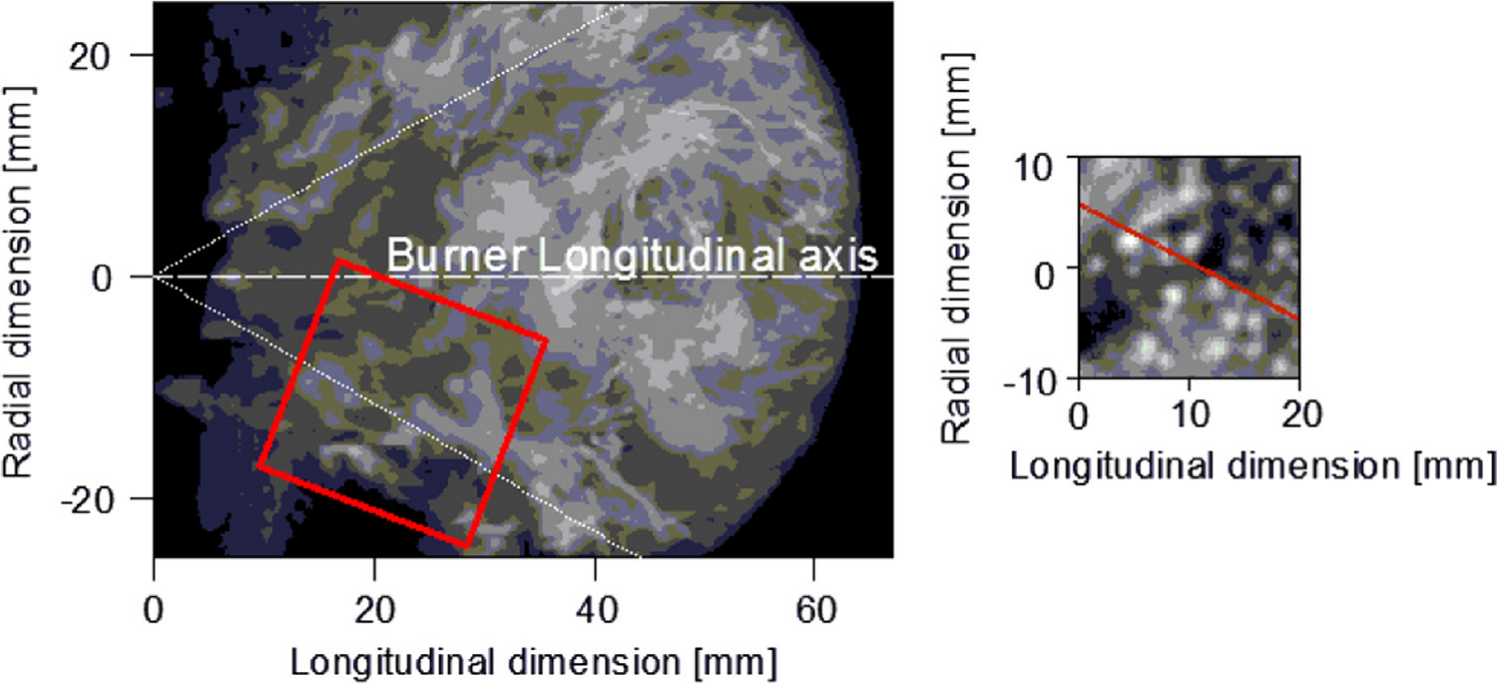
The picture on the left is an example of the flame image as acquired with MEMRECAM CCD, the red square is the area corresponding to the FLIR window and on the right an example of the flame image as acquired with FLIR CCD.

Visible images from the circumferential windows, on the left of Fig. 3, were taken with a resolution of 384×288 pixels and a frequency of 10 kHz. The electronic shutter was set to 3 µs and the time duration was 1 s for all the test conditions. A suitable flame view area of 67 mm (*h*) and 50 mm (*v*) was recorded during the acquisitions. The field depth is approximately 2 mm.

NIR images, as shown on the right of Fig. 3, were taken with a repetition rate of 1020 Hz and with a resolution of 64×64 pixels. The electronic shutter was set to 0.01 ms and the time duration was 1 s for all the test conditions. A suitable flame view area of 20 mm (*h*)x20 mm (*v*) was approximately recorded during the acquisitions. The depth of field is still approximately 2 mm.

Pollutant emissions (NO_*x*_, SO_2_, CO, CO_2_ and O_2_) were measured at the stack using a complete analyzer system (PG-350E Horiba) equipped with gas sampling, sample conditioning, analyzer and system control units [10]. The NO*_x_* detector uses the cross-flow modulation chemiluminescence detection system; the SO_2_ and CO detectors operate with a cross-flow modulation, non-dispersive infrared (NDIR) absorption method; the CO_2_ unit uses the standard non-dispersive infrared (NDIR) absorption method; and the O_2_ unit uses the paramagnetic method. The gas analyzer recorded the gas value every 1 s. The measurement uncertainties of NO*_x_*, CO, O_2_ and CO_2_ are less than ±1%. The measurement sensitivities are 1 ppm for NO*_x_*, CO and O_2_ emissions and 0.01% for CO_2_ emission.

The presented data refers to a total air flow rate (${\dot{m}}_{air}$) fixed in the range (58–64)·10^−3^ kg/s with a ratio of 1:11 between primary and secondary air flow respectively and a fuel flow rate (${\dot{m}}_{fuel}$) assuming the three values: 1.39, 0.93, and 0.58·10^−3^ kg/s. *Φ* assumes the three corresponding values: 0.333, 0.212, and 0.144.

The air was warmed up to 500 K. The pressure of the combustion chamber was set to 1 bar*_a_*.

Each variation of the fuel/air equivalent ratio was maintained for a 10 min interval, to ensure both stabilization of the equivalence ratio around the target value and steady state thermal boundary conditions.

## Footnotes

Appendix A Supplementary data associated with this article can be found in the online version at doi:10.1016/j.dib.2015.11.051.
